# Research translation mentoring for emerging clinician researchers in rural and regional health settings: a qualitative study

**DOI:** 10.1186/s12909-023-04786-0

**Published:** 2023-10-31

**Authors:** Olivia A. King, Alesha M. Sayner, Alison Beauchamp, Emma West, Drew Aras, Danielle Hitch, Anna Wong Shee

**Affiliations:** 1 Western Alliance, Warrnambool, Australia; 2 Monash Centre for Scholarship in Health Education, Clayton, Australia; 3 Deakin Rural Health, Deakin University, Warrnambool, Australia; 4 Grampians Health, Ballarat, Australia; 5 Monash University School of Rural Health, Warragul, Australia; 6 Victorian Heart Institute, Clayton, Australia; 7 Deakin University, Geelong, Australia; 8 Western Health, Sunshine, Australia; 9 Occupational Science and Therapy, Deakin University, Geelong, Australia

**Keywords:** Research capacity building, Research translation, Mentoring, Mentored training, Rural health, Clinician-researcher

## Abstract

**Background:**

Building clinician and organisation-level research translation capacity and capability is fundamental for increasing the implementation of research into health practice and policy and improving health outcomes. Research translation capacity and capability building is particularly crucial in rural and regional settings to address complex problems impacting these socially and economically disadvantaged communities. Programs to build clinicians’ research translation capability typically involve training and mentoring. Little is known about the features of and influences on mentorships in the context of training for emerging clinician-researchers working in rural and regional healthcare settings. Research translation mentorships were established as part of the Supporting Translation Research in Rural and Regional settings (STaRR) program developed and delivered in Victoria, Australia from 2020 to 2021. The study sought to address the following research questions: 1) What context-specific types of support do research translation mentors provide to emerging researchers?. 2) How does the mentoring element of a rural research translational training program influence research translation capacity and capability development in rural emerging researchers and mentors, if at all?. 3) How does the mentoring element of the program influence translation capacity and capability at the organisational and regional level, if at all?

**Methods:**

We conducted a qualitative descriptive study. Interviews with individuals involved in the STaRR program took place approximately 12 months after the program and explored participants’ experiences of the mentored training. Interviews were undertaken via telephone, audio-recorded, and transcribed. Data were analysed using a team-based five-stage framework approach.

**Results:**

Participants included emerging researchers (n = 9), mentors (n = 5), and managers (n = 4), from five health services and two universities. We identified four themes in the interview data: (1) Mentors play an educative role; (2) Mentoring enhanced by a collaborative environment; (3) Organisational challenges can influence mentorships, and (4) Mentorships help develop research networks and collective research and translation capacity.

**Conclusions:**

Mentorships contributed to the development of research translation capabilities. The capabilities were developed through mentors’ deepened understanding of the rural and regional healthcare contexts in which their emerging researchers worked, the broadening and strengthening of rural and regional research networks, and building and sharing research translation knowledge and skills.

**Supplementary Information:**

The online version contains supplementary material available at 10.1186/s12909-023-04786-0.

## Background

Research or knowledge translation refers to the dynamic and iterative processes used to implement research knowledge into healthcare practice and policy to improve healthcare delivery systems and outcomes [1–4]. Those leading research translation endeavours are often required to effect changes to established healthcare delivery systems and processes and are expected to influence the behaviours of multiple groups of healthcare professionals, support workers, and healthcare consumers. These groups of stakeholders may have different priorities, levels of power, authority, and influence [5], making research translation an inherently complex and non-linear process that must consider the characteristics of the local implementation context [3, 6, 7]. The burgeoning field of implementation science promotes the use of systematic and evidence-informed strategies to reduce the “knowledge-practice gap”, however, implementation science is still dominated by academic research and researchers [8, p. 332]. Targeted strategies are needed to build the capacity and capability of clinicians, program managers, and leaders in health settings to enact and support timely research translation [2, 9, 10]. In this context, capacity refers to the readiness, and access to infrastructure and other resources that individual and organisations require to engage in research and translation activity, whereas capability refers to the skills and competencies required to conduct research and translation endeavours [11].

Over the last five decades, programs and initiatives to enhance the research capacity and capability of health services and clinicians have been implemented in a bid to promote the conduct of applied, place-based health research [12–15]. By addressing contextually relevant factors in ways that consider the health delivery environment, approaches to embed research in health services aim to increase the timeliness, consistency, and sustainability of research translation and evidence-based practice [13, 16]. Healthcare needs and delivery is different for rural communities compared with urban settings. The low level of research funding for rural research [17] and the challenges the rural workforce face, such as chronic shortages, high rates of turnover and low levels of rural health professionals’ research capability and capcity, hinder the research capacity building needed for improving rural health outcomes [15]. Building the capacity and capability of rural and regional health clinicians to conduct, use, adapt, and translate research is therefore crucial to optimising the health of people living in these geographically dispersed and socially and economically disadvantaged communities [13, 18, 19].

Many of the programs to build research capacity and capability in health settings are multifaceted and comprise a mix of didactic learning, applied workshops, fellowships, and mentoring [12, 20]. The role of mentoring in the development of research skills in health clinicians is well-recognised [12] and is a feature of many programs that have an explicit focus on developing research translation skills [1, 2, 4, 13, 21–24]. Evidence describing the characteristics and functions of mentorships to build research translation and implementation science capacity in academic or research settings, has highlighted some of the key features and requirements of these mentorships. These include the need for mentors to be responsive, knowledgeable, and accessible [25], have altruistic motives, be honest and trustworthy, provide sound advice to their mentees [26], foster independence, and promote professional development [27].

In academia, research is more widely accepted as part of the role of the academic, and infrastructure and support are typically established around these expectations [28]. In the health service context, although research activity and the use of research to inform practice is recognised as central to healthcare delivery, there remain barriers to realising these practices. These barriers are well-documented and include time constraints [29], lack of knowledge and skills in research and translation [30, 31], and perceived or actual misalignments in organisational strategy and priorities [32]. Moreover, the complex and dynamic healthcare delivery environment can make research activity and translation difficult to achieve and sustain [2, 33]. For aspiring clinician-researchers based in rural and regional settings, these barriers are compounded by having fewer experienced researchers and networks to draw on as mentors to support their engagement in research [13, 34]. Mentoring is an effective mechanism to provide tailored support to novice clinician-researchers to navigate these challenges, and gain the confidence to engage in research and translation [4, 24, 35]. However, little is known about the features of and influences on successful mentorships for emerging clinician-researchers who embark on a research project within the rural and regional healthcare delivery setting. How these mentorships function, develop, and contribute to research translation capacity and capability building has not been explored in the health setting context [36].

### Study aims and research question

This study explored and described the impact of mentorships established as part of a research translation training program for rural and regional emerging clinician-researchers (emerging researchers hereon), and the influence of these mentorships on research translation capacity and capability building across the region. The study sought to address the following research questions:

1. What context-specific types of support do research translation mentors provide to emerging researchers?
2. How does the mentoring element of a rural research translational training program influence research translation capacity and capability development in rural emerging researchers and mentors, if at all?
3. How does the mentoring element of the program influence translation capacity and capability at the organisational and regional level, if at all?

### The STaRR program

In 2020, the Western Alliance Academic Health Science Centre [37], initiated the Supporting the Translation of Research in Rural and Regional Settings (STaRR) program for its member organisations which include nine health services, two universities, and one primary health network located in rural or regional settings in Western Victoria, Australia. Most of Western Alliance’s health organisation members are located in geographical areas that range from “regional centres” (Modified Monash category 2; situated within 20 km of a town) to those described as “small rural towns” (Modified Monash category 5) [38]. Prior to the implementation of the STaRR program, there were no region-wide coordinated research training programs in the area.

STaRR is a multifaceted program founded on the principles of research translation and aims to enhance the research and translation skills of rural and regional clinicians and build capacity within their health organisations to use and translate research into clinical practice and local policy. STaRR targets three key groups through training: research mentors (experienced academic or health service researchers), health managers, and emerging researchers. The inaugural STaRR program was implemented during the initial two years of the COVID-19 pandemic [39]. The STaRR program incorporated multiple pedagogies: experiential learning, social and collaborative learning, constructivism, and didactic learning, with a view to build emerging researchers’ self-efficacy [12, 40, 41]. See Table 1 for details about the participant groups, recruitment, and training.

**Table 1:** STaRR program participants and training

Role and definition	Function in STaRR program	Recruitment/engagement	Training format and topics
Mentors • Experienced academic or health service-embedded researchers • Located in universities and health organisations across the local region	• Support an emerging researcher for the duration of the STaRR program and potentially beyond • Support the STaRR program by developing and delivering training content or facilitating workshops activities	• Potential mentors were invited to submit an expression of interest (EOI) •EOIs were reviewed by the STaRR team for suitability (i.e., demonstrated experience in health services research or quality improvement) • Mentors were paired with emerging researchers by the STaRR team • Mentors were not remunerated for their mentor role	• Two 3-hour online workshops held two weeks apart • Prepared content, applied learning in small groups and discussions • Topics included an introduction to the STaRR program, the key concepts of research translation as informed by the TAHK framework, and the mentoring role within the STaRR program
Health managers • Senior clinicians or health program officers with decision-making authority • Located in health organisations (i.e., health services or a primary health network)	• Support research, translation, and building capability among their teams to engage in and use research in practice	• Health managers with an interest in research, translation, and building capability among their teams to engage in and use research in practice were invited to submit an EOI to participate in the STaRR Manager training • EOIs were reviewed by the STaRR team and appropriate candidates (i.e., those in a health service leadership role) were invited to participate in the training • EOIs submitted by less senior health service staff were invited to apply for the emerging researcher training	• Two 2.5-hour online workshops held two weeks apart • Prepared content and small and larger group discussions • Topics included the manager’s role in supporting research, collaborative research, ethics and governance requirements, supporting dissemination, and examples of health service-led research translation project were presented by health service manager peers
Emerging researchers • New or emerging clinician-researchers who identified a health practice or policy issue in their setting • Located in healthcare organisations	• Develop a protocol to conduct their translation-focused research project • Post-program: conduct research to address and local health practice or policy issue	• Aspiring STaRR emerging researchers submitted an expression of interest outlining an issue in local clinical practice or policy which could be addressed by research and translation • Submissions were developed in collaboration with, and signed by, participants’ managers to ensure support for their participation and alignment of the topic with their priorities • Emerging researchers were paired with a mentor by the STaRR program team based on their project ideas and workplace as they related to the mentors’ content or methodological expertise, and geographic location where possible	• Two-full day online facilitated workshops held five weeks apart • Prepared content, applied learning in small groups, and larger group discussions • Topics covered in the workshops included developing and refining a research question, research methodologies, developing a research translation team, ethics and governance requirements, and working with mentors • Mentoring occurred between and in numerous cases, beyond the two workshops, as emerging researchers were supported to develop a protocol to conduct their translation-focused research project

The mentoring element of the STaRR program was partially informed by the pre-existing Translating Allied Health Knowledge (TAHK) framework, which was co-developed with allied health clinicians [3]. The framework developed by Hitch and colleagues, comprises four domains: *doing* knowledge translation (KT), *social capital* for KT, *sustaining* KT, and *inclusive* KT. The term KT in this instance is synonymous with research translation.

Methods

## Methods

### Study design

This qualitative study is underpinned by social constructivism, a subjectivist epistemological position that privileges the meaning individuals make of their knowledge and experiences of the social world [42].

### Participant sampling and recruitment

With ethics approval (Barwon Health HREC, reference 20/183) all participants of the STaRR program from 2020 to 2021, including emerging researchers (n = 55), mentors (n = 27), and health managers (n = 32) were invited to participate in an interview via email. Interested participants were asked to read and sign a participant information and consent form prior to their interview.

### Data collection

Interviews were held between July and August 2022, approximately 12 months after the completion of the STaRR mentored training program for emerging researchers. Interviews were conducted via videoconference (Zoom), audio-recorded, and transcribed verbatim. An independent research consultant who was not involved in the development or delivery of the STaRR program conducted the interviews and asked questions about participants’ experiences of STaRR. The interview guide is presented in Additional File 1. Interviews lasted between 28 and 50 min (mean 36 min).

### Data analysis

Data were analysed using a five-step framework approach (Ritchie and Spencer, 1994). This involved five authors (AS, AB, EW, AWS, and OAK) familiarising themselves with the data and conducting cursory analyses of two or three transcripts each. These inductive analyses were used to develop an initial thematic framework. The coding framework was refined in consultation with the research team and was then used by one author (OAK) to code all the interview data via NVivo12. In consultation with the research team, patterns were identified within and across the data. Finally, these patterns were interpreted in the context of existing mentoring, research translation, and capacity building literature [12, 13, 15, 32, 43] and are reported in this paper.

### Researcher reflexivity

The team members involved in the data analysis process practiced a reflexivity exercise prior to commencing the data analysis. Through this collaborative exercise we identified varying levels of engagement and investment in the STaRR program and the influence this may have on our analyses. As the lead author and STaRR program manager, I continued to engage in reflexivity throughout the project reflecting regularly on my dual roles and the influence these had on the data collection, analysis, and interpretation processes. All research team members, including those without a direct role in the STaRR program contributed to the data analysis processes and reflected regularly on their experiences and perspectives in relation to the current study [44]. Prompts for the reflexivity exercise are presented in Additional File 2.

## Results

Participants represented three groups: emerging researchers (n = 9), mentors (n = 5), and managers (n = 4). Sixteen female and two male participants from eight geographic locations represented five rural and regional health services and two universities. Participants represented “metropolitan areas” (MM1; n = 6), “major regional centres” (MM2; n = 7), “large rural towns” (MM3; n = 1), “medium rural towns” (MM4; n = 2) and “small rural towns” (MM5; n = 1). One workplace location was missing. Participants were aged between 28 and 60 years old (median 40 years) and had between 3 and 36 years of experience in their current clinical, managerial or research role (median 15 years). The emerging researchers, mentors, and managers were recruited independently rather than as triads.

We identified four themes: (1) Mentors play an educative role; (2) Mentoring enhanced by a collaborative environment; (3) Organisational challenges can influence mentorships, and (4) Mentorships help develop research networks and collective research and translation capacity.

### Mentors play an educative role

Mentors provided different levels and types of support to their emerging researcher mentees so they can engage in the various research translation activities. Mentors invariably, facilitated experiential learning for their mentees by filling an educative role. Mentors often provided intense guidance to their emerging researcher mentees throughout the ethics submission process, which can be daunting for novice researchers:

She’s helped me work out some of the logistics around like data collection and what approval you need on patients’ data. . [Mentor] helped me quantify it and put it into the type of research we would be doing. . how we’ll store data securely. . She helped me a lot with writing the protocol. . I don’t think I would have got to that stage without her. Emerging Researcher 3.

On discussing expectations of the mentoring role from the mentor perspective, the extent of the educative function was at times greater than anticipated. Supporting clinicians’ research knowledge and skill development can be difficult in the clinical context, where, unlike in the academic supervision context, systems of teaching and learning research are not established:

I don’t think I realised it would be as intensive as the mentoring can be - it can be like trying to help someone get a masters through fortnightly mentoring, where I don’t have a curriculum to follow. . It can be very, very intensive and they need a lot of help because they don’t know what they’re doing a lot of the time. . it’s a teaching role at times. Mentor 3.

Emerging researchers also recognised and valued the educative role the mentors play, particularly around research methodology development, dissemination plan, and quality improvement and research implementation frameworks. Mentors provided guidance around the research activity as well as the application or implications of the findings:

We were meeting regularly to get her support with either all the team or parts of the team, just to ask her just all the questions. . It was a lot around research methodology and which [implementation] frameworks to kind of pick and, depending on what we were going to put into research and what we were going to keep as kind of quality improvement. . trying to figure out how we were going to tackle the project so that it could be published. Emerging Researcher 1.

Emerging researchers often described research as an overwhelming and unfamiliar process. Mentors were able to promote development of self-efficacy among their mentees by breaking down the research process into discrete and manageable activities and walking them through each of these:

[Mentor] was amazing support - right at the start with us, and then I feel like we’ve kind of – we’ve utilised everybody that we needed to. . just knowing that people are always there, that we can kind of tap on. . I feel like my brain couldn’t cope with all the information I needed to know for the whole project. So just knowing that I can just tackle it in bitesize pieces, and as we get to the next step. Emerging Researcher 1.

The quote above also highlights the role of the mentors in building upon the scaffolding constructed via the emerging researcher training workshops, and in promoting progress through what might otherwise be perceived as an insurmountable task. It also points to the reliance emerging researchers have on their mentors for support at various touch points, particularly in the absence of research supports within their organisations.

Mentors played an educative role as they guided the emerging researchers through the development and refinement of their research ideas and protocol and beyond. The level of support provided by mentors throughout these processes was recognised by both emerging researchers and mentors and led to a range of tangible research outcomes, and sustained capability development.

### Mentoring enhanced by a collaborative environment

The premise of the mentoring element was to work in concert with the STaRR training, research resources and support people across the region. The experiential nature of the STaRR training meant that emerging researchers were applying their learnings from the curriculum directly to a clinically relevant project with the guidance of their mentors:

Learning by doing makes it a lot easier for people to step into research, to have that support, to have a mentor, to have some direction along with the training. Without it, as a clinician, you’re always trying to meet your [clinical] targets. Emerging Researcher 8.

Participants described the value of combining the experiential learning with mentoring support to foster sustained engagement in research and skills development and longer-term engagement in research and translation:

STaRR focuses on a project - it’s designed that way to have a project in mind and at least work that project up into project protocol but ideally actually implement it. My experience is. . half don’t progress to that full project being done. However, all of that mentoring and discussing and supports and coaching and teaching fosters that within that clinical researcher. So, I expect to see the same people kind of come back in two years and say, ‘I’ve got another idea’. I think it does build capability and that’s a difficult output to measure. If you just looked at completed projects, it wouldn’t reflect that. Mentor 3.

The mentor above also noted not all participants will conduct a research translation project in its entirety within the STaRR program timeframes, and the limitations associated with only measuring the impact of research training and mentorship in the short-term. For mentors, the expectation of project completion as an outcome may be unrealistic and successful capacity and capability building can be measured in different ways.

Mentors were not expected to provide the full suite of support and guidance to their emerging researcher mentees, but rather draw on the wider pool of STaRR mentors and resources where possible and indicated. The next participant, described drawing on their networks including other STaRR mentors when appropriate, to ensure their emerging researcher has access to the right expertise at the right time:

What I’ve done is draw in others who also have expertise in a particular area or capacity to get involved as well. Had them take over as mentors where that suits better. . I think being able to be flexible, in terms of working with our network and perhaps seeking the most appropriate - someone who can provide additional mentorship, even if it’s perhaps less formal. Mentor 4.

The quote above highlights the value of a collaborative research and capability building environment, where mentors can support emerging researchers by drawing on their own networks.

The engagement and intentional inclusion of managers throughout the program was critical for the progress of the STaRR emerging researchers and their projects. There were examples of effective manager support, which helped secure resources, and aided the dissemination of information about emerging researcher-led projects:

Our managers have been really supportive of the project and have advocated to the executive for some additional funds to help us move this project along, because it is such an important area of practice. Emerging Researcher 5.

The manager described above was supportive and advocated for the emerging researcher, however this was not the case for all participants. This highlights the role for mentors in supporting and informing managers about their potential to advocate for and bolster their team members’ research endeavours.

Some emerging researchers found it challenging to identify relevant stakeholders and build and maintain relationships throughout their projects. Clinicians brought their knowledge of the rural and regional health context and then developed skills in appropriately engaging stakeholders through mentoring:

We’re looking at something that is cross disciplinary between [allied health disciplines] and also has medical and nursing as well. . and there’s a range of points from acute through to community. . the complexity of that and thinking about from an organisational perspective is quite challenging to know which staff we approach about it, in terms of various managers. . and clinicians. Emerging Researcher 6.

For many emerging researchers, the combination of their commitment, the sustained engagement with and encouragement of the STaRR program team, their mentors, and managers led to the achievement of research milestones, including dissemination of their research idea via conference presentations:

We put a submission in to present at the research symposium. . even though we haven’t started data collection they [STaRR Team and Mentor] were still encouraging of us putting in a submission. I wouldn’t have thought to do that before. . So, having those opportunities in a supportive space, where they just make you feel safe Emerging Researcher 4.

The emerging researcher quoted above also described how opportunities to share their work and connect with other “likeminded people” in a supportive environment, were important for their development. Mentorships existed within a collaborative research and learning environment, where mentors utilised their own networks to meet the needs of their emerging researchers. Managers were engaged to various extents throughout the program which influenced the progress of emerging researcher projects.

### Organisational challenges can influence mentorships

The three participant groups identified organisational factors as key influences on the progress of the emerging researcher-led projects and the research mentorships: namely time, funding, competing clinical service priorities, and the COVID-19 pandemic. Mentors, including those external to the health service setting, often understood the complexities of the healthcare environment. Although mentors were not able to ameliorate organisational and resource challenges, when they recognised the presence of these types of challenges, and took the opportunity to understand and impacts, they could better appreciate their emerging researchers’ capacity to progress their projects:

There absolutely are organisational factors. . I didn’t even really know this before this job, but there is just such a vast difference between a metropolitan teaching research hospital, which is a research institution in and of itself, so it has a department, a director, rooms, top software, teachers. If you kind of go all the way to the other end where you’ve got a rural hospital where of course research comes up, it’s still relevant, but none of that [infrastructure] exists. Mentor 3.

The mentor above also identifies the research infrastructure deficits evident in rural health settings, impeding research engagement. Mentors helped their emerging researchers to navigate some of these organisational challenges and resource deficits by working with them to first understand their organisational context, and then refine the scope and direction of the project to make it feasible:

[Mentor] had a really big impact on the idea development, and I guess project design as well, in the initial phases. . trying to make the project achievable within the constraints of our time and caseloads. So, we would have pretty regular meetings previously when we were in that development phase with [Mentor]. She would provide advice or just give us ideas to work within that space Emerging Researcher 5.

Similarly, mentors described their role in defining, and at times narrowing the scope of the projects being undertaken by their emerging researchers to develop a more achievable plan for progressing projects in light of the existing challenges in the rural and regional healthcare context:

I think [Emerging Researchers] might have been slightly over-ambitious about what they could potentially achieve with clinical load as well. So it was about sort of bringing them back down to earth about how much they could do and how many participants they would be able to recruit in that time as well. . I needed to, as a mentor, just be a little bit more realistic about how these projects work. Mentor 2.

Given the ongoing and widely experienced organisational challenges, emerging researchers and mentors discussed communication as being both critical to the mentorships, but at times, sub-optimal. The next mentor participant described feeling underutilised and unable to contribute to important decisions about the project due to poor communication:

Initially [we had] really good contact. . a very interesting discussion and then silence for quite a while. . the communication was very scant. . They might have been having conversations around, ‘is it the best timing? Have we the capacity to do it?’ But at no point was anybody sharing that with me. Mentor 1.

The ad hoc communication between emerging researchers and their mentors seemed to reflect the inconsistent nature of their research activity and progress due to competing priorities and other organisational challenges. It also reflected the different worlds in which the mentors and emerging researchers often occupied. Mentors were well-positioned to support their emerging researchers to navigate various challenges to engage in research, however this relied on effective and timely communication and genuine partnerships.

Emerging researchers, although cognisant of their need to drive communication with their mentors, described their appreciation of mentors checking in and sharing opportunities and relevant resources with them occasionally, to show they “weren’t forgotten”, even when they were not actively progressing their research due to competing priorities:

We were always driving it, but as soon as we reconnected, [Mentor] was straight back in there and sending things through, opportunities that might come up he’d always send through. . He just made us feel like we weren’t forgotten even when we weren’t actively working on the project. Just those subtle little, “here’s an opportunity”, or “I don’t know if you’ve seen this reference, or this resource”, was just really useful to kind of get back on track. Emerging Researcher 4.

The participant quoted above highlights the impact of mentors’ informal and ad hoc communications on keeping emerging researchers’ projects in their minds and on track.

Mentorships can be complicated by organisational factors impacting both mentors and their emerging researcher mentees. Effective, even if inconsistent communication, and mutual acknowledgement of these challenges can facilitate the development of project plans that account for such challenges. Mentors played a critical role in supporting emerging researchers to define and refine the focus of their projects and optimise the scope and achievability and sustainability of the research project in their organisational context and the challenges and constraints therein. They performed a motivational role at times by initiating ad hoc communication with their emerging researcher mentees at times they were disengaged from their research endeavours due to competing priorities.

### Mentorships help develop research networks and collective research and translation capacity

Research translation mentorships promoted the development of research networks across the region by facilitating the connection of emerging researchers, mentors, and managers with new contacts. This was particularly helpful for emerging researchers who were not engaged with research or researchers prior to their participation in the STaRR program:

My mentor also had lots of connections with universities which was really helpful and health services. . definitely being connected with other researchers and projects that are happening was helpful. . I wouldn’t have had any [other] way of finding out about [the research]. Emerging Researcher 8.

The quote above exemplifies the isolation rural and regional clinician-researchers can experience. Similarly, the next emerging researcher valued building relationships with others across their region to reducing professional isolation, progressing research and translation endeavours, and to thinking about their research translation problems in new and different ways:

Networking is a really big thing. . we’re not the only healthcare service that are dealing with these types of things. . we definitely found that that was really useful to get different people’s perspectives and to see who else is in the region as well. . the STaRR Program definitely helped for me getting my foot in the door, to be able to build relationships with some very amazing researchers in the area. Emerging Researcher 1.

Mentoring in the rural and regional context lends opportunities for mentors to develop their relationships with and knowledge of other researchers and their research endeavours, providing pathways for subsequent collaborations:

One of the advantages of being a rural and regional focused program is that we get to know people. We know who in the region might be working on this or who has expertise in this. As part of the mentor-mentee program, we’ve been able to get to know them and what they do better. There’s been a couple of instances of people then going onto work together in another project afterwards. Mentor 4.

Mentors described drawing on their networks for support and motivation as they embark on what can be a long mentorship journey with their emerging researcher mentees:

What enables me as a mentor is the network that we have of us all really as mentors, formally or informally, to keep each other – the positivity and keeping the goal in mind and a recognition that this is a long game. Mentor 3.

With respect to capability building, the emphasis was often on the emerging researcher, with little or no attention to the capability building and support needs of mentors. The need to bolster the mentor network to build collective understanding of the mentor role, how the mentorships are functioning, and seek support from one another was identified:

[the mentor network] does exist, they just don’t necessarily know who’s out there or what’s expected of them or how our roles can do that. So, we did try things like a Slack [Communication Platform]. . chatroom type thing and it doesn’t necessarily need to be something like that. . I just imagine that some mentors feel a bit adrift once they’re paired up and it’s all happening. Mentor 3.

Mentors spoke of the benefits from learning about other projects happening across the region throughout the STaRR program and mentoring process. This next mentor described utilising their networks and knowledge of other projects across the region, to link their emerging researcher mentee into:

I think just getting to know additional work that’s happening in the region and being able to link up others in the region with mentees has been a really great experience. . being across what they’re [emerging researchers] doing, where they work and what their goals are. If that overlaps with some over work that might be happening in the region or another part of my role, I’ve been able to make an introduction and try and establish a relationship that’s mutually beneficial. Mentor 4.

The quote above demonstrates how the mentoring process strengthened and expanded research networks, social capital, and potential for collaboration across the region for both mentors and their emerging researcher mentees. It also points to the mutually beneficial learning and development opportunities afforded in mentorships.

Engaging in the STaRR program also provided managers with new contacts and mechanisms to seek early feedback on a project idea, which may influence the progress of research within their teams:

. . all of that [new project development] has been helped by doing the STaRR program, seeing what others are doing and then also having those networks and connections where I’ve been able to talk to people and get feedback. Two weeks ago, we met with [Research Mentor] and [Embedded Researcher] and got some feedback on our very broad project plan and it was really useful. Manager 1.

Emerging researchers who participated in the STaRR mentored training were able to share their learnings and skills with team members in their local setting, and contribute to other projects, therefore extending and sustaining the impact beyond the individual to teams and organisations. This suggests there are opportunities to develop emerging researchers as peer mentors throughout the mentored training and experiential learning processes:

We’ve got a couple of different projects going within our speech pathology team and the project that I’m working on at the moment is with the dietetics team as well. So, I think coming through the program I’ve been able to contribute and help upskill in some other areas as well in terms of more around the project idea development and the protocol side of things Emerging Researcher 5.

The mentorships facilitated the development of research and translation networks across the three participant groups, which represent both emerging and experienced researchers, working across academic and health service settings, and health managers in decision-making positions. Additional and tailored support for mentors to engage in productive mentorships and build their own skills and networks is needed to ensure the success and sustainability of the mentoring aspect of the STaRR program.

## Discussion

The research and translation capacity and capability-building literature indicates the need for multiple combined strategies to promote experiential learning, with mentored training among one of the most well-documented strategies [12]. This qualitative descriptive study aimed to describe the context-specific types of support research translation mentors provided to emerging clinician researchers participating in a rural and regional research translation training program; how the mentoring element of the program influenced the development of research translation capacity and capability in individual emerging researchers and mentors; and at the organisational and regional levels, if at all. The activities, tasks, and outcomes described in this paper are specific to the mentorships established for clinician-researchers based in rural and regional healthcare delivery settings and are predicated on a translation-focused research project, rather than general career or skill development as described in the existing research translation mentoring literature [26, 45]. Our findings indicate enhanced individual, organisational, and regional capacity and capability [43] through mentors’ deepened understandings of the rural healthcare context, the strengthening and broadening of research networks, and the development and sharing of new research and translation knowledge and skills. See Table 2 for a summary of the key findings.

**Table 2:** Key findings

• A **collaborative environment** cultivated by the rural and regional research translation training program provided an ideal context for mentorships to support emerging clinician-researchers
• Mentors took opportunities to deepen their understanding of the rural and regional healthcare context within which their emerging clinician-researcher mentees worked and tailored their approaches and the outcomes they anticipated according to the **organisational challenges**
• Mentors provided instrumental support and played an **educative role** for their emerging researcher mentees, which was at time intensive and taxing for the mentors
• Research translation mentorships contributed to the expansion and strengthening of **rural and regional research networks** for emerging researchers, mentors, and health managers
• Experienced researchers willing and able to fulfil the mentoring role for rural and regional emerging clinician-researchers are a valuable and finite resource
• Mechanisms to support, engage, retain, and grow the rural and regional mentor group are needed

The combination of the workshops underpinned by experiential pedagogies, constructivism, peer learning, and the mentoring between and beyond the training workshops created a collaborative environment that facilitated the development of research and translation skills. These skills were developed in the context of well-documented health organisational factors, challenges, and time constraints related to competing priorities [2, 7, 13, 46]. In academic supervision models and many clinician-researcher mentoring programs, mentors are often co-located with or situated in similar academic or clinical research workplace settings as their mentees [47]. The mentors in the current study were frequently situated externally to their emerging researcher’s rural or regional healthcare workplace. Importantly, mentors took opportunities to understand the context within which their emerging researcher mentees worked in, and the challenges impacting their research progress and capability development [46].As such, mentors provided support that accounted for and addressed these challenges (e.g., by narrowing the scope of the project or breaking projects down into manageable and discrete activities, and helping to identify key stakeholders to support the project). Mentors were able to support emerging researchers to align the scope and direction of their projects with the organisational context and priorities, which was key to ensuring project support and sustained research translation [3]. These findings echo those of Gagliardi et al. [4] on the importance of health service-based knowledge users’ need for tailored, context-specific, and timely knowledge translation mentoring. Further, this points to the need for mentors to develop a deepened understanding of the health service and rural and regional healthcare contexts to work effectively with their emerging researchers. This reciprocity in learning facilitates both health service and academic organisational and individual capacity and capability development [48].

Facilitating connections between and across the three groups: emerging researchers, mentors, and managers, led to the expansion and strengthening of research networks [25, 49], and contributed to the development of individual, organisational and regional research translation capacity and capability. Strengthening the networks of health researchers in rural and regional settings, where there are fewer and typically geographically dispersed health researchers than in metropolitan areas, is critical to reducing professional isolation and achieving a critical mass of rural researchers [32, 34, 35, 50]. Building research networks is frequently cited as one of the mentees’ aims and outcomes of mentorship [51]. In previous research, the development of networks has rarely been a focus for mentors. Where mentors had access to others in the network with specific skills, knowledge, and connections, they felt confident to provide their emerging researcher mentees with a more comprehensive level of support, and also enhanced their own research capability. This research reinforces the value of engaging in research mentorships for both emerging researchers and mentors, particularly in rural and regional training programs. It also points to the need to integrate mechanisms to engage, support, and build further mentors’ capabilities and networks [52]. Although mentors in the current study did not explicitly describe the need for incentives to drive their engagement in, and contributions toward the program, these must be considered in future programs [36, 45].

The STaRR program mentorships focused on building the capacity and capability at multiple levels of influence within health services, which is pivotal for sustaining capability building and research translation in these organisations [3, 9]. Engaging managers throughout the program led to some teams securing funding for additional time and resources to dedicate to the project to make their completion feasible [9, 41, 53, 54]. Furthermore, there were examples of managers having access to mentors who provided ad hoc research support, which was an unintended consequence of the mentoring element, and highlights the expanded reach of the program through mentors’ deeper engagement with the health services. However, not all managers of emerging researchers were engaged in the STaRR program and not all had access to mentoring support. This indicates that among other strategies to build research translation capability in managers such as targeted training, [53] and team-based interventions involving managers [9], there may be role for mentors in providing dedicated, tailored support to managers to operationalise and embed research and translation in rural and regional health settings.

Building individual-level research translation capability was achieved in part through extended mentorships beyond the training workshops that helped to consolidate emerging researchers’ newly developed knowledge and skills [1, 24] and to address additional capability building needs identified over time. Mentors provided several types of instrumental support to their emerging researcher mentees [47] including fulfilling an educative role to supplement and reinforce the training content which provided “scaffolding” for their emerging researcher mentees [40, 55]. Specifically, mentors helped to decipher and select appropriate research methodologies, refine the research question, and secure funding and other resources needed to progress the projects. Mentors also provided psychosocial support through ad hoc communication about research events, encouragement to pursue opportunities, or generally bolstering their confidence [26, 47]. This support helped their emerging researcher-mentees to maintain a level of engagement with their research projects amid competing priorities. These types of communication and engagement are particularly crucial for rural and regional emerging clinician-researchers who are geographically and often, professionally isolated [56]. Furthermore, mentors helped their emerging researchers identify suitable implementation frameworks, which is a recognised challenge for clinician-researchers engaging in research translation [2], and important to consider early on to promote the successful and sustained translation of findings into practice [57]. For some mentors however, the intensive instrumental support required was taxing and unsustainable, particularly in the absence of an academic framework or curriculum. Given the finite number of experienced health researchers who are willing and able to mentor rurally based emerging researchers, the sustainability of mentored research training programs and retention of skilled mentors must be considered [34, 58]. This is particularly the case where there is no guarantee of a research output, such as a peer-reviewed publication, which is a key performance indicator for academic researchers [59] and often an intended outcome of academic supervision [60, 61]. Research led by emerging clinician-researchers in rural and regional settings is often hindered by challenges related to small clinical teams or solo clinicians, higher staff turnover, and chronic workforce shortages [34, 56, 62]. This means rural and regional clinicians may need to occupy multiple roles and outcomes such as evidence-informed practice change and research publications often take longer to achieve [59].

Mentorships were often characterised by frequent, albeit ad hoc communication and tended to produce tangible outcomes in terms of research translation progress. Some mentors in our study, however, felt underutilised and disregarded due to the inconsistent and inadequate communication. The need for clarity of expectations and ongoing negotiations around commitment to ensure productive and sustainable mentorships are well-recognised [25, 26, 45, 48] and may also promote ongoing mentor engagement and retention. The findings of the current study suggest that the markers of success for rural and regional research translation mentorships need to be carefully considered [48], given the resource and other constraints impacting clinician-researchers working in these often geographically and professionally isolated settings [15].

### Limitations

This study is limited by the small proportion of participants representing each of the participant groups. The study is set in a geographical context that comprises a mix of rural and regional health organisations and may not reflect the geographic or population profile of metropolitan or more remote and resource-constrained contexts. Further, the participants representing the three groups were not necessarily related to one another or working on the same project, therefore the dynamics of each of these triads were not investigated in the present study. The self-selecting participants may have had more engagement with and better experiences of the program. Notwithstanding, numerous interview participants reported ceasing their engagement either as an emerging researcher or mentor and contributed to data about potential modifications to enhance the STaRR program. The mentorships explored in this research were established in the context of a mentored training program, therefore the findings of this study are applicable to similar programs, rather than mentorships as a standalone initiative. Nonetheless, mentored training programs are a commonly utilised research capability building strategy [12]. The participant sample was more female dominant than the average health workforce. Future research ensuring a sample more representative of health and health services research workforces is needed.

The research team members had varying levels of involvement with the program, from leading its development and delivery, to no direct engagement. It is possible that those with direct and sustained involvement in the program delivery introduced a level of bias to the study. Notwithstanding, internal evaluations can have advantages over those conducted externally, including an in-depth understanding of the program elements, contexts within which the participants learn and work, and familiarity with the colloquial language used by the participants. Moreover, the engagement of independent researchers in the data collection and analysis processes helped the research team maintain a reflexive approach [63]. Finally, the interviews followed the completion of the inaugural STaRR program and did not account for the longer-term outcomes that may have since been realised. There is a clear need for longer-term investigation of mentorships and the impacts of these, including potential ripple or unintended outcomes and impacts.

## Conclusions

We have identified some of the key features of research translation-focused mentorships between emerging researchers working in rural and regional health settings and experienced academic or health services researchers and mechanisms that influenced research translation capacity and capability building. Mentors took the time to understand the context within which their emerging researcher mentees worked and tailored their mentoring approach and expectations accordingly. Mentors drew on their own networks to enhance their research translation capabilities and provide comprehensive support for their emerging researcher mentees. In turn, mentors developed their own knowledge and networks and those of their emerging researcher mentees. Mentors supported manager engagement throughout the STaRR program, which was imperative and contributed to organisational research translation capacity and capability building. However, managers work within the same challenging environments as emerging researchers and strategies to build their capacity and capability to operationalise research and translation, including mentoring and coaching, must be targeted. Mentors performed an instrumental and educative role to reinforce and supplement the scaffolding set up via the training content, and support emerging clinician-researchers to develop focused and achievable research translation projects in rural and regional health settings. Strengthening networks and continued engagement with the STaRR program is expected to continue to grow regional research translation capability, however this relies on the ongoing commitment, retention, and growth of the mentor group. The sustainability of the STaRR and similar mentored research training programs requires intentional efforts to understand mentors’ capability building needs, and their desired outcomes.

### Electronic supplementary material

Below is the link to the electronic supplementary material.

Supplementary Material 1

Supplementary Material 2

## Data Availability

Additional qualitative data collected and analysed as part of the current study, which is not published in this article, are not publicly available due to participant confidentiality but may be made available from the corresponding author on reasonable request.

## List of abbreviations

EOI: Expression of interest
KT: Knowledge translation
STaRR: Supporting Translation of Research in Rural and Regional Settings
TAHK: Translating Allied Health Knowledge
