# Supplementary material for: Research translation mentoring for emerging clinician researchers in rural and regional health settings: a qualitative study
Source: BMC Med Educ. 2023 Oct 31;23:817. doi: 10.1186/s12909-023-04786-0 (PMC10617223; doi:10.1186/s12909-023-04786-0)
Supplement: Supplementary file 1 — Supplementary Material 1 [file 12909_2023_4786_MOESM1_ESM.docx]

## Additional File 1 Semi-structured interview guide

Background

You have had some involvement with the STaRR program however as a reminder, the region-wide initiative involves:

- *three research translation coordinator roles covering the [anonymous] areas and a number of organisations to support healthcare practitioners with research translation;*
- *a mentored training program for healthcare practitioners (this involves training for mentors and new and emerging-researcher practitioners), and*
- *research strategy support workshops with organisations guided by consultants*

This interview will in part, contribute to our evaluation of the whole program.

Are there any questions before we get the interview started?

**Interview guide for practitioners**

Q1. Please introduce yourself, your role and where you work

Research skills

Q2. Where are you at in terms of progressing your research project?

Q3. Please tell me about your experience of the STaRR Program

*How have your skills in research changed since you participated in the STaRR program?*

*What (if anything) have you found valuable about the program?*

Q4. Which research skills in particular, that you gained from the training, have been useful and applicable to your research and other work (e.g., clinical practice or program development)?

Q5. Which skills do you need to develop (through training or other means) to increase your capacity to conduct research?

Mentoring

Q5. What was the mentoring element like for you?

*Are you still working with your mentor?*

*What worked well?*

*What could be improved in future?*

*What have been the main contributions (if any) your mentor has made to the development of your project?*

*What have been the main learnings or skills you have developed (if any) since working with your mentor? This could relate to research OR clinical practice.*

*How has your mentor supported your access to different forms of knowledge (e.g., networks or resources you were not previously aware of)?*

*What skills or qualities did you value most in your mentor?*

Organisational and other support

Q6. What organisational factors have influenced the progress of your research?

Q7. What other factors (if any) have influenced the progress of your research?

Q8. What role has the Western Alliance Research Translation Coordinator at your health organisation played throughout your research experience, either before, during, or after your participation in the STaRR program?

*Please provide any examples of when your Research Translation Coordinator has supported you to use evidence to guide practice, progress a research activity, or connect you with people or resources so you can progress*

Research translation

Q9. How has the STaRR program influenced research or research translation in your organisation?

*Describe any examples*

*Has the Research Translation* *Coordinator played a role in research translation? [this may include planning for translation of your findings into practice]*

Q10. Do you have any other comments, ideas or thoughts about research translation or research capacity building, that you have not had the chance to express?

**Interview guide for mentors**

Q1. Please introduce yourself, your role and where you work

Mentoring

Q2. To the best of your knowledge, how has your mentee’s project progressed since they completed the STaRR training program?

*Did the mentee/s’ research progress as you had expected?*

Q3. What has your experience as a mentor been like?

*What worked well?*

*What could be improved in future for both mentors and mentees?*

*What qualities did you value most in your mentee?*

Q4. Reflecting on your experience as a mentor, were there any unexpected learnings, challenges or opportunities that arose?

*Learnings*

*Challenges*

*Opportunities*

Organisational and other factors

Q5. From your perspective, were there any organisational factors that influenced the progress of the mentee/s’ research?

*Enabling factors*

*Constraining factors*

Q6. What role do you think the Research Translation Coordinator played in the health service at which your mentee worked?

*Did you cross-over or interact in any way while you were supporting your mentee?*

*Have you had any other experiences working with any of the Research Translation Coordinators? If so, please describe*

Training

Q7. Do you think that the RT training was appropriate in terms of content and delivery, for the new and emerging researchers, to start them on their research journey?

*What worked well?*

*What could be improved in future?*

Q8. Are there any additional training needs for mentors to be addressed?

Research translation

Q9. The STaRR training is aligned with the Translating Allied Health Knowledge (TAHK) Framework. Have you applied the TAHK framework in practice? If so, please tell me about a scenario where you have applied it. *This may not be related to the STaRR program, but another project you have been involved in.*

Q10. From your perspective, how has the STaRR program influenced research or research translation in your organisation or across the region?

*What are (or could be) the markers of success?*

Q11. Do you have any other comments, ideas or thoughts about research translation or research capacity building, that you have not had the chance to express?

**Interview guide for health organisation managers**

Q1. Please introduce yourself, your role and where you work

Q2. How have your research or research translation knowledge or skills changed since you participated in the STaRR program?

*How have your knowledge or skills influenced the way you manage or lead your team, or plan your operational work?*

Q3. Did any of your team members participate in the STaRR training? *If so, please describe any changes or improvements you have noticed in their research skills or the way they approach clinical practice problems.*

Q4. Thinking about your organisation and its strategic direction, how is research and research translation described or espoused?

Q5. Have you or your team members had any contact with your organisation’s Research Translation Coordinator? If so, please provide details of the contact and any support provided.

Q6. How has the STaRR program influenced research or research translation in your organisation or the region?

*Examples of any outcomes or impacts?*

Q7. How can regional research and research translation be further strengthened through the STaRR Program or other means?

Q8. Do you have any other comments, ideas or thoughts about research translation or research capacity building, that you have not had the chance to express?
