# Supplementary material for: Research translation mentoring for emerging clinician researchers in rural and regional health settings: a qualitative study
Source: BMC Med Educ. 2023 Oct 31;23:817. doi: 10.1186/s12909-023-04786-0 (PMC10617223; doi:10.1186/s12909-023-04786-0)
Supplement: Supplementary file 2 — Supplementary Material 2 [file 12909_2023_4786_MOESM2_ESM.docx]

**Additional File 2 Qualitative data analysis reflexivity exercise**

**Name:**

**Research project:** Translating Research in Rural and Regional Health Settings

1. **Why did I start the research in the first place?**
2. **What clinical or other experience have I had that may influence the research?**
3. **How might my clinical or other experience influence the research?**
4. **What theories may apply to, or support the research (if any)?**
5. **What do I expect to come out of the research?**
6. **What do I hope to get out of the research?**

Barry, C. A., Britten, N., Barber, N., Bradley, C., & Stevenson, F. (1999). Using reflexivity to optimize teamwork in qualitative research. *Qualitative Health Research*
